# Re-validation and cultural adaptation of the brief, standardized assessment tool for measuring HIV-related stigma in healthcare settings in Almaty, Kazakhstan

**DOI:** 10.1371/journal.pone.0276770

**Published:** 2022-11-02

**Authors:** Balnur Iskakova, Zhamilya Nugmanova, Recai Murat Yucel, Kristi E. Gamarel, Elizabeth J. King

**Affiliations:** 1 Department of Epidemiology, School of Public Health, Kazakh National Medical University Named After S.D. Asfendiyarov, Almaty, Kazakhstan; 2 Department of Epidemiology and Biostatistics, Temple University College of Public Health, Philadelphia, Pennsylvania, United States of America; 3 Department of Health Behavior and Health Education, School of Public Health, University of Michigan, Ann Arbor, Michigan, United States of America; Nazarbayev University School of Medicine, KAZAKHSTAN

## Abstract

The HIV epidemic continues to grow in Kazakhstan and HIV stigma remains a major barrier to HIV prevention and treatment in the country. HIV stigma in healthcare setting may also discourage people living with HIV (PLHIV) from getting the care needed. Therefore, studying the attitudes of healthcare workers towards PLHIV is important and requires well-constructed measurement tools adapted to the specific cultural context. In our study, we aimed to adapt and re-validate a brief questionnaire on HIV stigma among healthcare workers in Almaty, Kazakhstan. We held focus group discussions to obtain input on an existing questionnaire and surveyed 448 primary healthcare providers to psychometrically evaluate the scale. The final HIV-stigma scale consisted of 15 items, 6 of them measuring negative opinions about PLHIV and the rest assessing stigmatizing health facility policies towards PLHIV. Both HIV-stigma subscales demons6trated adequate psychometric properties (with Cronbach’s alpha α = 0.57 for the first and α = 0.86 for the second subscale, and with factor loadings >0.35 within each subscale). High numbers of respondents holding negative attitudes towards PLHIV, detected in this sample (87%; n = 380), may suggest the need for immediate actions addressing HIV stigma in healthcare in Kazakhstan.

## Introduction

Human immunodeficiency virus (HIV)- related stigma continues to be a barrier to addressing the HIV epidemic, restricting access to prevention, testing and treatment services for those who need the services the most [1]. Discriminatory behaviors towards people living with HIV (PLHIV), as a manifestation of stigma, has been linked to poorer psychological wellbeing among individuals affected by HIV, which can result in social isolation and decreased retention in HIV care [2–5]. HIV stigma in healthcare settings can serve as an extra burden for PLHIV in getting necessary medical care [3].

Kazakhstan, a country in the Eastern European and Central Asian (EECA) region, is gradually meeting the goals set by the Joint United Nations Program on HIV/AIDS on ending AIDS by 2030 (95-95-95). According to the latest estimates, around 77% of PLHIV in Kazakhstan (among men and women aged 15 years and older) were aware of their status, 57% were on antiretroviral therapy and only 48% had suppressed viral loads by 2020 [6]. HIV stigma has been posited to be one of the main contributing factors to low levels of HIV care coverage in the country [7–10]. A survey conducted among PLHIV in several regions in Kazakhstan showed healthcare facilities to be the most commonly reported setting of experienced HIV stigma and discrimination: 17.6% of the respondents reported receiving some levels discrimination and 6% of them suggested strong discriminative behaviors from healthcare staff [5].

Despite the significance of HIV stigma in each step of the HIV care continuum, there are numerous limitations in addressing this issue scientifically. The measurement of HIV stigma has been largely restricted to the perspectives of PLHIV [11], which neglects to account for the role of those who enact stigma. In other words, a focus on PLHIV has the potential to place the responsibility on those being stigmatized to cope with discrimination without attending to the structures and systems that perpetuate these conditions. Studying HIV-related stigma from the perspective of those who enact is also important in attempting organization-level interventions on addressing this issue [12].

The validity of HIV stigma scales being developed presents an additional challenge. Systematic reviews suggest numerous scales are being used, many of which are not validated or adapted to different languages and cultures [13, 14]. Translating a measuring instrument into the language of the study population alone has shown to be not adequate for its further use [15, 16]. This is particularly true if the phenomenon is an attitude that cannot be measured and compared across cultures directly [15]. In addition, country specific characteristics of the phenomenon under investigation need to be considered within the adaptation of survey tools. For example, PLHIV are generally known to face multiple stigmas interlinked between HIV-related stigma and other forms of marginalization such as gender identity, sexual orientation, occupation and drug abuse history. This issue is particularly relevant to countries where traditional values and norms are confronted with such phenomena [16, 17].

There is no study that addresses the challenges of measuring HIV-related stigma in a Kazakhstani context in our understanding. Therefore, this exploratory study is aimed to re-validate the brief HIV stigma assessment tool [18] in Kazakh and Russian languages and adapt it to country-specific characteristics of the HIV epidemic.

## Methods

### Adaptation process

The brief HIV stigma assessment tool used in this study was designed and validated in multiple diverse country settings (China, Dominica, Egypt, Kenya, Puerto Rico and St. Christopher & Nevis). It was specifically developed for healthcare workers in medical facilities, including both clinical and non-clinical staff [18]. The initial structure of the questionnaire comprised of 22 items divided into 5 sections focused on actionable causes of HIV stigma and discrimination: background information, infection control, health facility environment including policies, and opinions about PLHIV.

Considering the specific characteristics of the HIV epidemic in Kazakhstan, we modified the current tool by adding items from a previously validated Ethiopian questionnaire on HIV stigma and discrimination [19]. Attitudes towards sexual identity, sex work and drug abuse are rooted on cultural and religious believes and strictly regulated by legal instruments in both countries. The items included from the Ethiopian tool were to detect the differential opinions about PLHIV based on a mode of HIV transmission (sexual intercourse, drug injection and blood transfusion) and feeling of shame due to one’s HIV-positive status. Other additional items include opinions about HIV-positive patients’ plans on having children since PLHIV were likely to be advised not to have children by healthcare professionals or rarely receive adequate information on healthy pregnancy in earlier surveys [5].

The brief HIV- stigma assessment tool was previously translated into Russian by another research team and used in Kazakhstan. We have used this previous translation of the questionnaire for Russian speaking respondents with a mutual agreement between two research teams. The Kazakh version of the questionnaire was translated from English by the main study investigator and translated back into English by an independent expert.

#### Focus group discussion (FGD)

The questionnaire with added items was pilot tested first and discussed within a session of FGD prior to the main surveys. The FGD was conducted to discuss the clarity and relevance of all of the items both in Kazakh and Russian languages and lasted for thirty minutes. The discussion group included a general practitioner, a nurse, a social worker, and a psychologist.

The findings from the FGD discussions revealed an unfamiliarity with some of the study terms such as “Men who have sex with men or MSM”. The need for additional explanations for such terms prior to the main survey was therefore noted. Another issue raised was the misinterpretation of the study items in “Fear of HIV transmission at work” section (e.g., «How worried would you be about getting HIV if you drew blood from a patient living with HIV?”). These items were designed to capture the general fear of contracting HIV at work regardless of having or not having an HIV-positive patient during the study period. However, increasing numbers of nurses during the pilot study responded “not applicable” to a question on concerns of contracting HIV during the usual medical procedures that most of them practice. Therefore, we added a clarifying sentence as “How worried would you be about getting HIV if you did the following? Regardless of the presence of HIV-positive patients at the moment”.

### Validation process

The final survey included socio-demographic information and revised HIV-related stigma items (Table 1).

**Table 1:** HIV-related stigma measures in three study languages.

Items	English version	Russian version	Kazakh version
**Section 4** **Health facility policies**	**Do you strongly agree, agree, disagree, or strongly disagree with the following statements?**	**Вы согласны, полностью согласны, не согласны или категорически не согласны с перечисленными ниже утверждениями?**	**Сіз төмендегі мәлімдемелермен келісесіз, толығымен келісесіз, келіспейсіз немесе толығымен келіспейсіз, сәйкесін таңдаңыз**
Item 15	In my facility it is not acceptable to test a patient for HIV without their knowledge	В моем учреждении не допускается тестировать пациента на ВИЧ без его ведома.	Мен жұмыс жасайтын мекемемде науқасты АИТВ-на оның білуінсіз тексеруге жол берілмейді
Item 16	I will get in trouble at work if I discriminate against patients living with HIV.	У меня будут проблемы на работе, если я буду вести себя дискриминирующим образом в отношении людей, живущих с ВИЧ	Менің тарапымнан АИТВ-мен өмір сүретін адамдарға қатысты кемсітушілік көрсетілген жағдайда менің жұмыс орнымда қиыншылықтар туындайды
Item 17 (added)	I will get in trouble at work if I disclosure a patients’ HIV status to others without her or his consent	У меня будут проблемы на работе, если я раскрою статус пациента, живущего с ВИЧ, другим без его или ее согласия	Мен АИТВ-мен өмір сүретін науқастың статусын басқаларға оның келісімінсіз жариялаған жағдайда менің жұмыс орнымда қиыншылықтар туындайды
Item 18a	There are adequate supplies in my health facility that reduce my risk of becoming infected with HIV	В моем медицинском учреждении достаточно средств, снижающих риск моего заражения ВИЧ	Менің медициналық мекемемде, менің АИТВ-ні жұқтыру қаупін азайтатын шаралар жекілікті
Item 18b	There are standardized, procedures/protocols in my health facility that reduce my risk of becoming infected with HIV.	В моем медицинском учреждении внедрены стандартизированные процедуры / протоколы, снижающие риск моего заражения ВИЧ	Менің медициналық мекемемде, менің АИТВ-н жұқтыру қаупімді азайтатын стандартталған рәсімдер / протоколдар енгізілген
Item 19	My health facility has written guidelines to protect patients living with HIV from discrimination	В моем медицинском учреждении внедрены оформленные в письменной форме руководящие указания, направленные на защиту людей, живущих с ВИЧ, от дискриминации	Менің медициналық мекемемде АИТВ-мен ауыратын адамдарды кемсітуден қорғауға бағытталған жазбаша түрдегі жетекшілік нұсқаулары енгізілген
**Section 5. Opinions about PLHIV**			
Item 20a	Most people living with HIV do not care if they infect other people.	Большинство людей, живущих с ВИЧ, не волнует вопрос, а что, если они заразят других людей.	АИТВ-мен өмір сүретін адамдардың көбісін олардың АИТВ-н басқа адамдарға жүқтыру мәселесі толғандырмайды
Item 20b	People living with HIV should feel ashamed of themselves	Людям, живущим с ВИЧ, должно быть стыдно за себя	АИТВ-мен ауыратын адамдар өздері үшін ұялуы керек.
Item 20c	Most people living with HIV have had many sexual partners.	У большинства людей, живущих с ВИЧ, было много сексуальных партнеров	АИТВ-мен өмір сүретін адамдардың көпшілігінде бірнеше жыныстық серіктері болған
Item 20d	People get infected with HIV because they engage in irresponsible behaviors.	Люди заражаются ВИЧ при безответственном поведении	Адамдар АИТВ-н жауапсыз мінез-құлықтары арқасында жұқтырады
Item 20e	HIV is punishment for bad behavior.	ВИЧ–это кара за неправильное поведение	АИТВ-ы—бұл дұрыс емес әрекеттер үшін берілген жаза
Item 20f (added from the Ethiopian scale)	I would feel ashamed if someone I know got HIV/AIDS.	Мне было бы стыдно, если бы кто-то из моих знакомых был ВИЧ инфицированным	Егер менің таныстарымның арасында АИТВ-н жұқтырған адам бар болса, мен ол үшін ұялатын едім
Item 20g (added from the Ethiopian scale)	I would feel ashamed if someone in my family got HIV/AIDS.	Мне было бы стыдно, если бы кто-то в моей семье был ВИЧ инфицированным	Егер менің отбасымның мүшесі АИТВ-н жұқтырған болса, мен ол үшін ұялатын едім
Item 20h (added from the Ethiopian scale)	HIV positive patients who acquired the virus through sexual intercourse are more at fault for contracting HIV than those who got it by blood transfusion	ВИЧ-инфицированные пациенты, которые заразились вирусом при половом акте, больше виноваты в заражении ВИЧ, чем те, кто заразились при переливании крови	АИТВ-н жыныстық қатынас арқылы жұқтырған науқастар, вирусты жұқтырғанына, қан құю кезінде жұқтырған науқастарға қарағанда көбірек кінәлі.
Item 20i (added from the Ethiopian scale)	HIV positive patients who acquired the virus through drug injection are more at fault for contracting HIV than those who got it by blood transfusion	ВИЧ-инфицированные пациенты, которые заразились вирусом при инъекции наркотиков, больше виноваты в заражении ВИЧ, чем те, кто заразились при переливании крови	АИТВ-н иньекциялық есірткі арқылы жұқтырған науқастар, вирусты жұқтырғанына, қан құю кезінде жұқтырған науқастарға қарағанда көбірек кінәлі
Item 21	Women living with HIV should be allowed to have babies if they wish.	Женщинам, живущим с ВИЧ, должно быть разрешено иметь детей, если они этого хотят.	АИТВ-мен өмір сүретін әйелдерге, егер олар қалаған жағдайда, балалы болуға рұқсат берілуі керек

#### Socio-demographic variables

Participants were asked their age, gender (male/female), position (e.g., physician, dentist, nurse), ethnicity, religious affiliation (including religiousness), years of work in healthcare in general, experience of working with PLHIV (e.g., “Among your patients in the past 12 months, did you have any patients who you knew to be HIV-positive?”), and training on HIV and HIV-stigma related issues (e.g., “Did you ever receive training in HIV-related stigma and discrimination”?).

#### HIV stigma scale

The HIV stigma scale included in this study contains 15 items. Six of them assessing stigmatizing health facility policies (e.g., “My health facility has written guidelines to protect patients living with HIV from discrimination.”) and nine items addressing negative opinions about PLHIV including the patients with different modes of HIV transmission history such as sexual contact, drug injection and blood transfusion (e.g., “HIV positive patients who acquired the virus through sexual intercourse are more at fault for contracting HIV than those who got it by blood transfusion.”). The response options to the “Opinions about PLHIV” items were on a 4-point Likert scale ranging from “Strongly agree” to “Strongly disagree” while the items on stigmatizing health facility policies included “yes”, “no”, and “not sure” categories. Total stigma scores were created by averaging item responses on both subscales for testing the data for normality.

#### Convergent and divergent validity variables

HIV- related stigma variables and a set of social-demographic items were used to conduct convergent and divergent validity analysis. The overall percentage of people holding stigmatizing attitudes towards PLHIV was calculated from the items 20A,20 B, 20D and 21 in ‘Opinions about PLHIV’ following the guidelines provided by the original scale developers [18]. The following responses can be considered as stigmatizing based on these guidelines: agreeing at least with one of the stigmatizing statements such as “People living with HIV should feel ashamed of themselves”, “Most people living with HIV do not care if they infect other people” and “People get infected with HIV because they engage in irresponsible behaviors’ or disagreeing with allowing HIV positive women to have children if they wanted to. The overall percentage of the respondents with stigmatizing opinions towards PLHIV was coded as “yes stigma” and “no stigma” for further use in the analysis. Socio-demographic data used for convergent and divergent validity analysis included years of work (categorized as >4 years, 5–15 years and more than 15 years), self-identified religiousness (measured on a Likert scale from “highly religious” to “not religious at all”) and whether the responded had seen an HIV-positive patient within the last 12 months.

### Data collection

The research team first contacted the chief medical officers of the eight randomly chosen primary healthcare clinics (polyclinics), out of 65 available, in Almaty for recruitment purposes. Next, employees of the clinics were invited to complete the survey. Participation was voluntary and clinic administration was not aware of who did or did not choose to complete the survey. Eligible participants were 18 years of age or older, fluent in Kazakh and/or Russian languages, and had at least 1 year of work experience in healthcare. In total, 448 healthcare providers including both clinical and non-clinical staff were eligible to participate.

After completing the recruitment process of study participants, a set of cross-sectional surveys were conducted in 8 polyclinics in Almaty from May 2, 2019 to July 2, 2019. The questionnaires were made available in Kazakh and Russian languages for a choice during surveys. Conference halls provided at each study setting were used to conduct the self-administered surveys during morning and evening shifts. A study investigator was also available throughout the survey time to answer any questions, specifically, with the terminology that was raised within the pilot study. Ethical approval for this research was received from Kazakh National Medical University Ethics Committee (IRB session №5/82). Written informed consent forms in Russian and Kazakh languages were provided to respondents before completing the survey.

### Data analysis

One way frequency tables were generated to provide descriptive statistics. We conducted an exploratory factor analysis (EFA) from a randomly selected sample of approximately half of the participants (*N* = 268), using SPSS. A principal component analyses for categorical data or CATPCA (based on optimal scaling method) was applied to test how well the newly added items fit within hypothesized factors. Factor loadings of more than 0.35 were considered as high enough to keep the items in the questionnaire. Internal reliability for the subscales was assessed with Cronbach’s alpha and the cutoff value of 0.60 was used to determine the internal consistency of the items. We then used R [20] to conduct a Confirmatory Factor Analysis (CFA) on the 15-item scale from the other half of the participants (n = 180) who did not overlap with the EFA sample. We evaluated the goodness of fit with a chi square test with corresponding degrees of freedom. However, chi square model fit criterion is known to be highly sensitive to large sample sizes and can lead to erroneous conclusions [21]. Therefore, we also evaluated the comparative fit index (CFI), and the root–mean–square error of approximation index (RMSEA). A CFI score of ≥0.90 is generally considered indicative of an acceptable fit. RMSEA values between 0.06–0.08 indicate an acceptable fit, with higher values indicating a need in improving the fit [22]. Missingness in the total dataset ranged from 3.3% to 10%, depending on the item and were not included in factor analysis.

Additional analyses were conducted in this study to report convergent and divergent validity. We fit multivariable logistic regression models that statistically adjusted for age, gender, and clinical staff. We hypothesized more years of work experience and seeing patients living with HIV would be associated with stigmatizing attitudes (for convergent validity). We also hypothesized that self-identified religiousness might exhibit divergent validity and have no links to stigmatizing attitudes. Significance level of alpha<0.05 was used to determine the associations within these models.

## Results

### Sample characteristics

The demographic characteristics of the sample can be found in Table 2. The majority of participants were female (92%, n = 413) and nurses (62%, n = 274). The predominant number of female workers in the sample is expected due to the homogeneous (mainly female) structure of the most healthcare settings in the country. Participants’ age ranged from 19 to 74 (M = 40.02, SD = 13.92). Most of the participants were of a Kazakh ethnicity 81% (n = 359), and 83% (n = 366) of the sample identified themselves as Muslims. Only one fifth of the study sample (18%, n = 79) reported receiving some training on HIV-related stigma and discrimination, including discrimination towards key affected populations.

**Table 2:** Sociodemographic characteristics of the survey sample (n = 448).

Variables	N (%)
**Gender**	
Male	8% (35)
Female	92% (413)
**Age group**	
18–29	31% (138)
30–40	17% (78)
41–51	22% (97)
>52	30% (135)
**Religion**	
Christian	8% (36)
Islam	82% (366)
Judaism	1% (3)
Not religious	6% (26)
Other	2% (10)
Missing	1% (7)
**Ethnicity**	
Kazakh	80% (359)
Russian	8% (34)
Uighur	5% (20)
Ukrainian	1% (6)
Other	5% (22)
Missing	1%(7)
**Professional category**	
Doctors/Physician	22% (99)
Dentist	3.5% (16)
Nurse	62% (274)
Psychologist/Social worker	4% (19)
Cleaning staff	4% (19)
Other	3% (14)
Missing	1.5%(7)

### Construct validity analyses

HIV-stigma scores were approximately normally distributed with the mean scores ranging from 1.26 (SD = 0.51) for opinion section to 0.21 (SD = 0.08) for health policy section. The Kaiser-Meyer-Olkin (KMO) test and Bartlett’s test, conducted before the EFA to test the factorability, revealed the KMO value of 0.78 and a statistically significant Bartlett’s test of sphericity (p<0.001) meaning that EFA can be applied to the obtained dataset. Exploratory factor analysis from the data with the randomly selected half of participants suggested that two-factor solution that corresponds with the items that assess “stigmatizing health facility policies’ and ‘negative opinions about PLHIV’. Factor retention was decided by examining eigenvalues, scree plot, and interpretability of factors, which all suggested a two-factor solution (with total eigenvalue of 7.14 with 48% of variance explained, and with eigenvalues for each corresponding factors: 4.91 (33%), 2.23 (15%)). Factor loadings within each factor were above the cutoff value (0.35), ranging from 0.43 to 0.81 within each subscale (Table 3).

**Table 3:** Factor loadings of the original and extended HIV-related sigma scales on EFA models (N = 196).

Item content by factor	Factor loadings	
**Factor 1: ‘Health facility policies’**	**1**	**2**
Item 15	-0.145	0.456
Item 16	0.159	-0.426
Item 17	0.144	-0.570
Item 18A	-0.299	0.708
Item 18B	-0.242	0.776
Item 19	0.299	-0.364
**Factor 2: ‘Opinions about PLHIV’**		
Item 20A	0.587	0.145
Item 20B	0.759	0.220
Item 20C	0.750	0.082
Item 20D	0.721	0.115
Item 20E	0.785	0.173
Item 20F	0.782	0.227
Item 20G	0.734	0.250
Item 20H	0.681	-0.048
Item 20I	0.615	-0.219

CFA models demonstrated rather contradictory results: χ^2^ = 239.47, *p*<0.001, CFI = 0.95, TLI = 0.94, SRMR = 0.12 indicating good fit while RMSEA values suggesting poorer model fit (0.11). Model revision with modification indices revealed largest MI values for the item 20H (51.89) in “Opinions about PLHIV” section (e.g., “HIV positive patients who acquired the virus through sexual intercourse are more at fault for contracting HIV than those who got it by blood transfusion”) and the item 20I (e.g., “HIV positive patients who acquired the virus through drug injection are more at fault for contracting HIV than those who got it by blood transfusion”) (S1 Table and S1 Fig). Another highest MI (43.63) was seen for the items 20F (e.g., “I would feel ashamed if someone I know got HIV/AIDS”) and 20G (e.g., “I would feel ashamed if someone in my family got HIV/AIDS”). Similar wordings used in items may cause such correlated errors within CFA models as suggested by the literature [23, 24]. In other words, there might be a systematic variance shared by these items which is not related to the factor. Two correlated error terms included within the second CFA model, which can be theoretically justified by above-mentioned variables having similar item wordings, have significantly improved the model’s fit (S2 Table and S2 Fig). The final CFA model revealed acceptable goodness-of fit when assessed by GFI (0.97), TLI (0.97), and RMSEA (0.07) making it an appropriate measurement model for the 15-item instrument. As illustrated in Table 4, items loaded significantly onto the two-factor model and the CFA model demonstrated acceptable and good psychometric properties of the subscales (Cronbach’s alphas for factor 1 was α = 0.66 and α = 0.85 for factor 2).

**Table 4:** Standardized factor loadings with corresponding standard errors of the original and extended HIV-related scales on CFA models (N = 159).

Item content by factor	Factor loadings
**Factor 1: ‘Health facility policies’**	
Item 15	0.38 (--)
Item 16	0.63 (0.53)
Item 17	0.46 (0.41)
Item 18A	0.85 (0.56)
Item 18B	0.92 (0.63)
Item 19	0.50 (0.35)
**Factor 2: ‘Opinions about PLHIV’**	
Item 20A	0.46 (--)
Item 20B	0.64 (0.19)
Item 20C	0.67 (0.21)
Item 20D	0.58 (0.19)
Item 20E	0.76 (0.23)
Item 20F	0.86 (0.24)
Item 20G	0.79 (0.24)
Item 20H	0.79 (0.24)
Item 20I	0.67 (0.21)

Note: Dashes (--) indicate the standard error was not estimated.

### Convergent and divergent validity

Convergent and divergent validity multivariable analysis demonstrated significant associations between years working and stigmatizing attitudes such that those who worked 5–15 years had a reduced odds reporting stigmatizing attitudes compared to those who worked less than 5 years (AOR = 0.33, 95% CI: 0.12, 0.84, *p* = 0.02). There was also significant association between seeking a patient living with HIV and stigmatizing attitudes such that those who reported seeing a patient living with HIV in the past 12 months was associated with a reduced odds of stigmatizing attitudes (AOR = 0.34, 95% CI: 0.18, 0.62, *p*<0.001). In contrast, there was no significant association between self-identified religiousness and stigmatizing attitudes.

## Discussion

The current study is aimed to re-validate the brief assessment tool on HIV-related stigma in healthcare and to adapt it to Kazakhstani healthcare settings. The results from our data analysis have demonstrated that it is possible to extend the HIV-related stigma scale for adaptation purposes and for further use in Kazakh and Russian languages. This study also has allowed us to better understand HIV-related stigma in healthcare demonstrating considerably high levels of negative opinions (overall percentage of people holding stigmatizing attitudes) towards HIV-positive patients by almost 87% (n = 380). This is considered to be one of the immediately actionable causes of HIV-stigma and needs to be addressed accordingly [25].

The HIV-stigma scale with added items in this study demonstrated good psychometric properties. Cronbach’s alphas for each HIV-stigma subscales ranged from 0.57 to 0.86 demonstrating acceptable to excellent internal consistency. The lower Cronbach’s alphas for the subscale 1 can be explained by the reduced variance that derives from the use of 3-item responses within “Health facility policies” section. The original validation study of the scale demonstrated similar results with Cronbach’s alpha level of α = 0.78 for the combined sample of six countries [18]. There are few other studies that attempted to develop a standardized assessment tool on HIV-stigma among healthcare providers and have proven its feasibility [26, 27]. However, none of the studies were conducted in Kazakhstani healthcare settings reporting the psychometric properties of the translated tools to our best knowledge. It adds an extra challenge in future use of such measurement tools, questioning the clarity of the translations and relatability of the study items to the local characteristics of the HIV epidemics.

HIV/AIDS-related stigma can be experienced as an additional burden to already existing stigmas associated with specific groups and behaviors such as queer, sex workers, drug users and people involved in casual sex. These issues were addressed in the original tool by including the items on refusals of providing care to the HIV key populations [18]. The current study attempted to refine these items by asking questions on negative attitudes towards PLHIV based on the mode of HIV-transmission. Adding these items in “Opinions about PLHIV” did not affect the high reliability of the scale seen within the original study. As these items also demonstrated, over a third of the respondents agreed with the statement that HIV-positive individuals with a history of sexual transmission and drug abuse are guiltier of their HIV-positive status than those who have acquired the infection otherwise. This suggests a concerning level of differential attitudes towards PLHIV based on a mode of HIV transmission.

There are few key lessons to be learned within the validation process of the survey tool in this study. One of the issues within implementation of the surveys was the unfamiliarity of study respondents with skip pattern items. The authors of the original brief questionnaire suggested the use of iPads or other electronic devices in order to make the skip pattern responses automatic and to provide more privacy to the respondents [18]. This study applied a paper and pencil method and additional clarifying explanations were provided within the questionnaire (e.g., ‘22a. If I had a choice, I would prefer not to provide services to people who inject illegal drugs’ with the following clarifying question such as ‘I agree with the above mentioned statement in 22.a because:..’). Other comments made over the questionnaire were the complexity of some of the translated items in Kazakh language which were then simplified by the study investigators prior to the main surveys.

This study is the first of its kind in re-validating the brief HIV-related stigma tool in Kazakh and Russian languages applying a mixed method approach to data collection and analysis. However, there are some limitations to be discussed. We considered only two dimensions of HIV-stigma from the original scale due to the remaining methodological challenges in other sections. The clarifications made over the “Fear of HIV transmission at work” items (“How worried would you be about getting HIV if you did the following? Regardless of the presence of HIV-positive patients at the moment”) were insufficient in this study. Considerable numbers of respondents answered “not applicable” to these items that needed to be treated later as missing affecting the sample size required for factor analysis. The exclusion of missing data from factor analysis is another limitation to be discussed. Multiple imputation methods (MI) applied failed to yield credible inferences due to unique challenges pertaining to rates of missing data across a large number of variables. Estimates such as rate of missing information and added variance due to missing data were much larger than the rates of raw missing data, indicating that the models used to sample missing data were not sufficiently informed by the observed data, and thus they were not identifiable. Finally, our principal component analysis for the EFA is based on conventional assumptions and does not assume random errors. As this is a limitation of the analysis, we note that the impact of such assumption to be minimal on the component scores which might be used for classification or regression, and that the real impact might be on the variability in the component loadings [28]. Therefore, our findings on the factor loading might be highly variable should the random errors with high variances exist, which might not be the case as the data collection was done in a face-to-face fashion.

## Conclusion

The aim of this study was to re-validate the HIV-related stigma tool in two languages spoken in Almaty, Kazakhstan. As it was seen on FGDs in this study, adapting the translated tools to the local study setting was is important to prevent any misinterpretations of HIV-stigma items. This study also demonstrated that it is possible to modify the existing scales in order to capture the most relevant information related to HIV-stigma in Kazakhstani context. This scale may later be used in understanding the potential sources of such negative attitudes towards PLHIV applying more sophisticated statistical analysis. In addition, immediate actions on high levels of negative opinions towards PLHIV in primary healthcare settings are urgently needed.

## Supporting information

S1 Table Modification indices CFA model 1. (TIF)

S2 Table Modification indices CFA model 2. (TIF)

S1 Fig Path diagram CFA model 1. (TIF)

S2 Fig Path diagram CFA model 2. (TIF)

S1 Dataset (CSV)

S2 Dataset (SAV)

S3 Dataset (CSV)
