# Supplementary figures and images for: Re-validation and cultural adaptation of the brief, standardized assessment tool for measuring HIV-related stigma in healthcare settings in Almaty, Kazakhstan
Source: PLoS One. 2022 Nov 2;17(11):e0276770. doi: 10.1371/journal.pone.0276770 (PMC9629601; doi:10.1371/journal.pone.0276770)

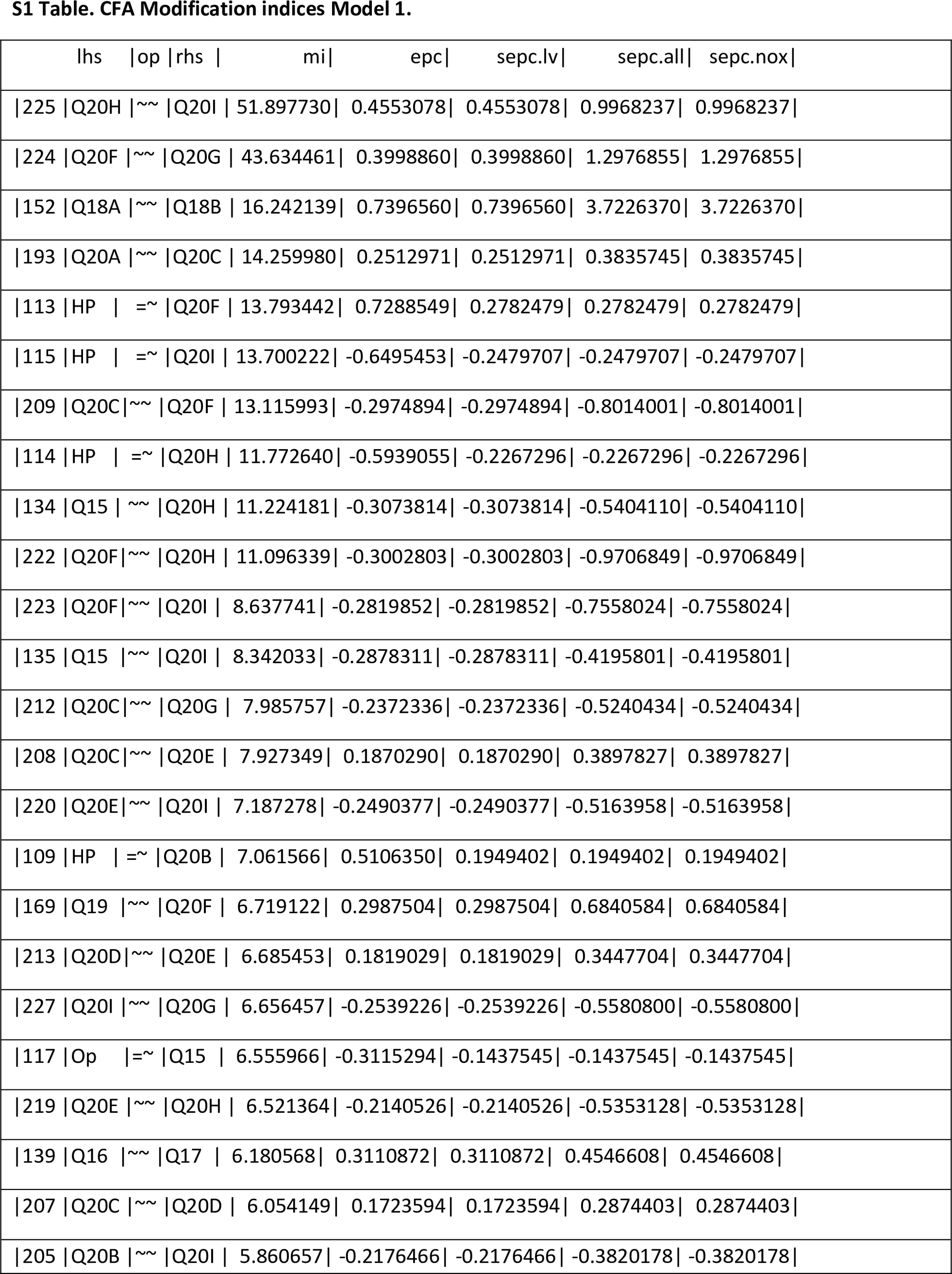

Supplement: S1 Table — (TIF) [file pone.0276770.s001.tif]

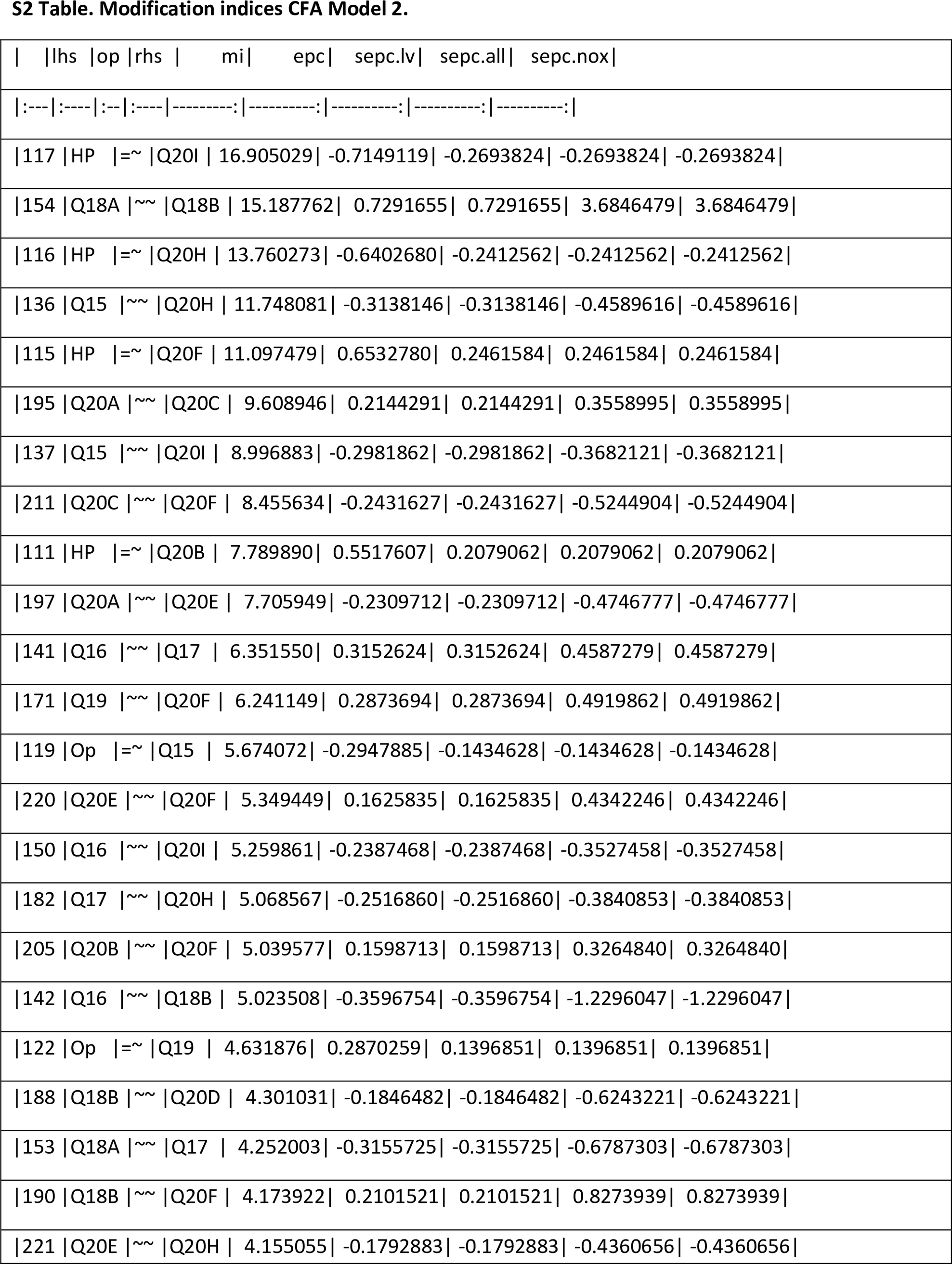

Supplement: S2 Table — (TIF) [file pone.0276770.s002.tif]

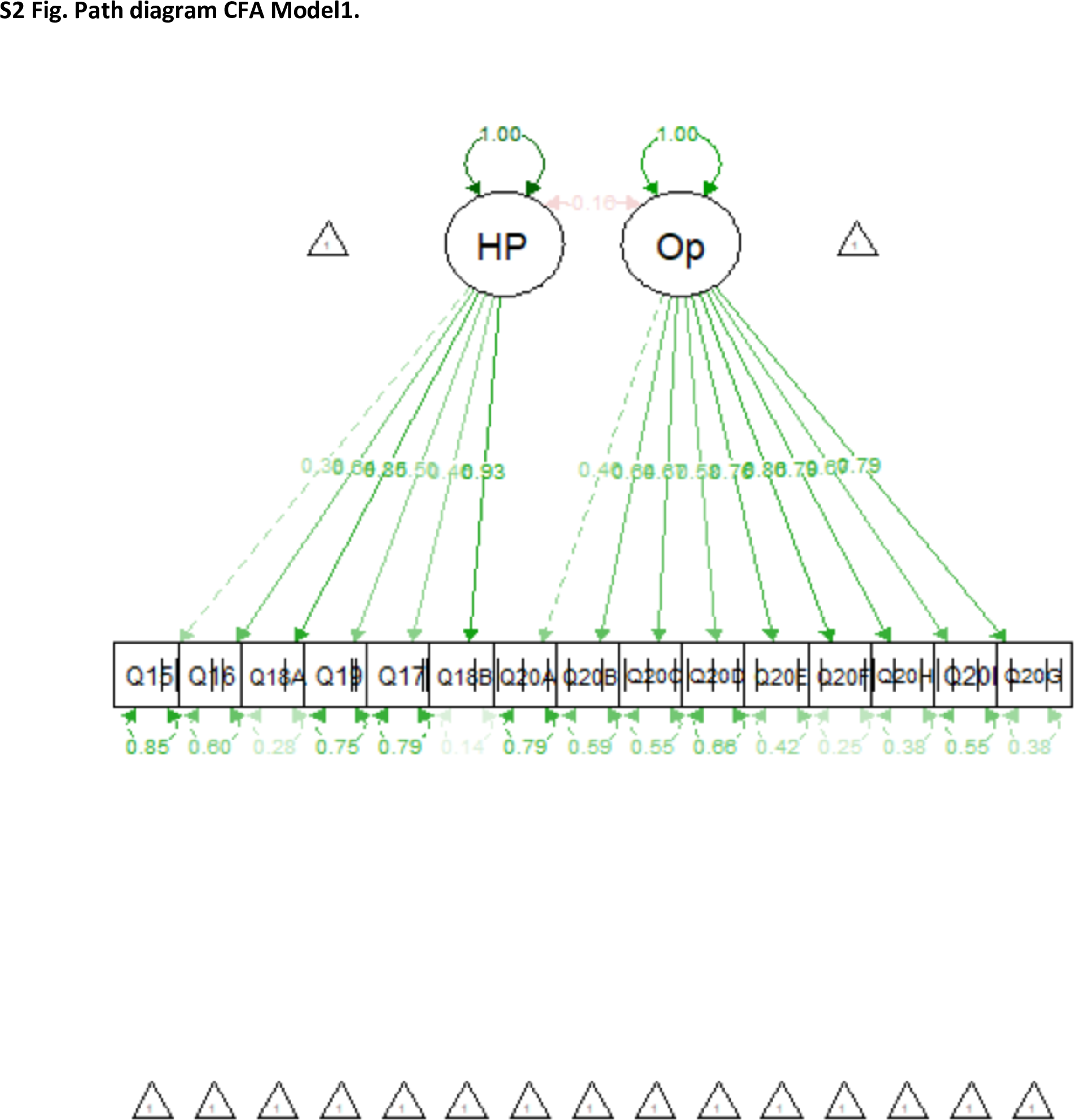

Supplement: S1 Fig — (TIF) [file pone.0276770.s003.tif]

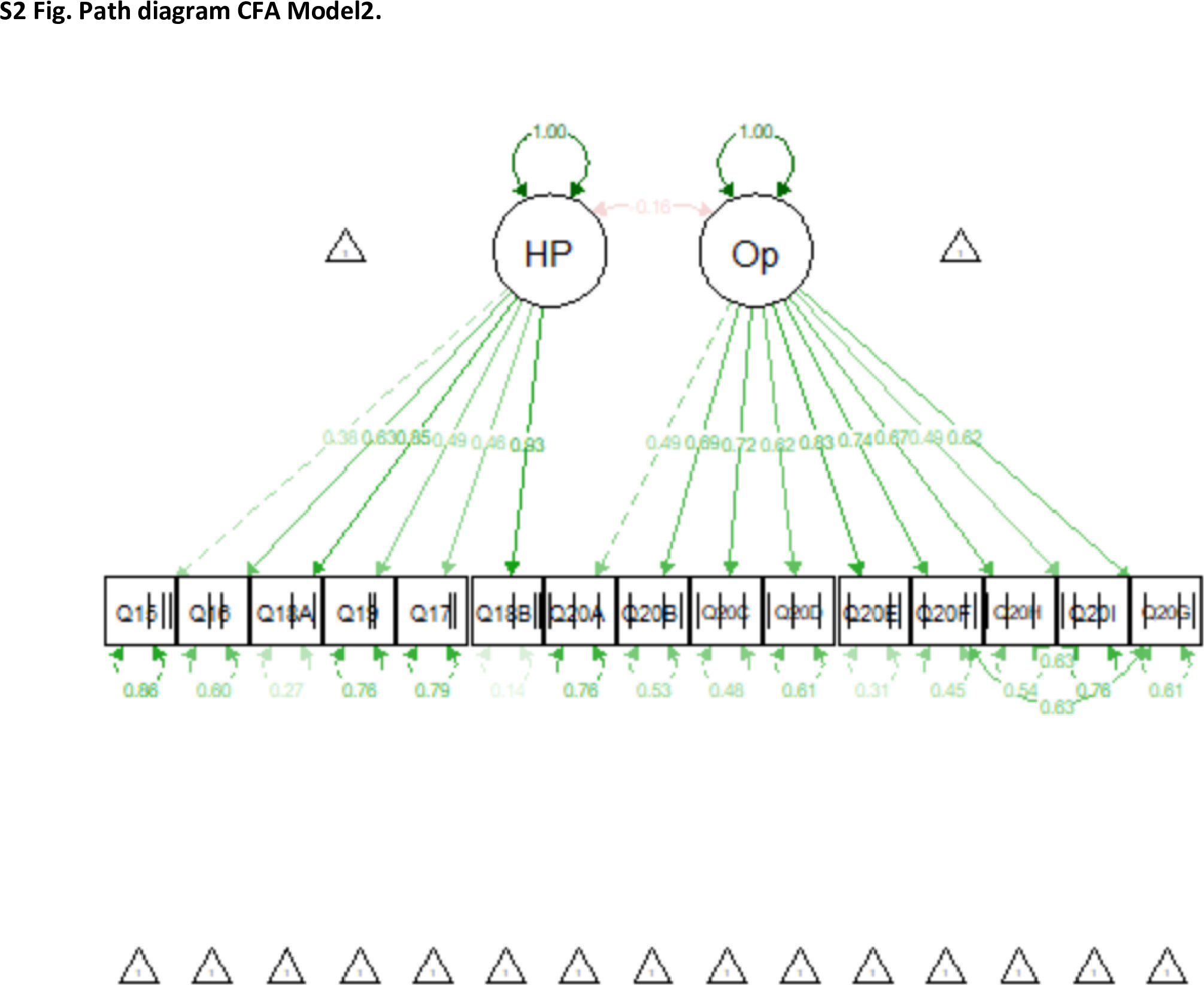

Supplement: S2 Fig — (TIF) [file pone.0276770.s004.tif]
